# Endoplasmic Reticulum Stress-Related Factors Protect against Diabetic Retinopathy

**DOI:** 10.1155/2012/507986

**Published:** 2011-12-10

**Authors:** Wei-Kun Hu, Rong Liu, Han Pei, Bin Li

**Affiliations:** Department of Ophthalmology, Tongji Hospital, Tongji Medical College, Huazhong University of Science and Technology, Wuhan 430030, Hubei Province, China

## Abstract

The endoplasmic reticulum (ER) is a principal mediator of signal transduction in the cell, and disruption of its normal function (a mechanism known as ER stress) has been associated with the pathogenesis of several diseases. ER stress has been demonstrated to contribute to onset and progression of diabetic retinopathy (DR) by induction of multiple inflammatory signaling pathways. Recent studies have begun to describe the gene expression profile of ER stress-related genes in DR; moreover, genes that play a protective role against DR have been identified. P58^IPK^ was determined to be able to reduce retinal vascular leakage under high glucose conditions, thus protecting retinal cells. It has also been found by our lab that ER-associated protein degradation factors exhibit significantly different expression patterns in rat retinas under sustained high glucose conditions. Future research based upon these collective genomic findings will contribute to our overall understanding of DR pathogenesis as well as identify potential therapeutic targets.

## 1. Introduction

**Figure 1:**
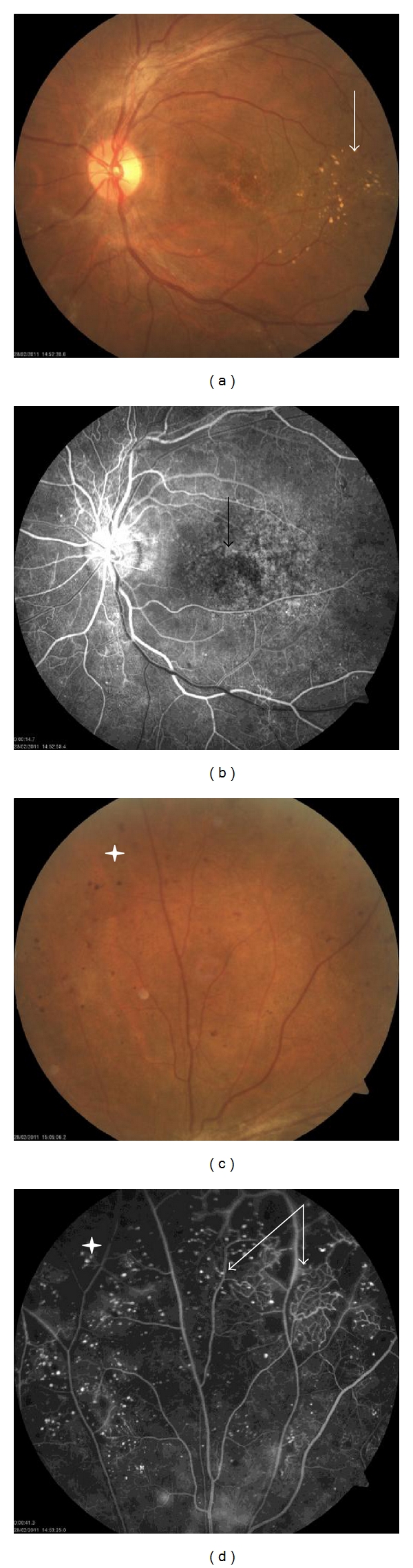
Fundus photographs (a, c) and retinal fluorescence angiography (b, d) of a patient with proliferative diabetic retinopathy. (a, b) and (c, d) are images of the same part of the retina. The Fundus photographs revealed widely scattered spots on the retina, which represent microaneurysms (white star in c). In addition, we observed that the macular foveal reflex had disappeared. Yellow-white exudates were apparent on the temporal area of the macula (white arrow in a). The angiograms were obtained during the arterial phase (b) and the late arteriovenous phase (d), after injection of dye into an antecubital vein. Retinal neovascularization was observed adjacent to areas of vascular nonperfusion (white arrow in d). The multiple, tiny fluorescent dots (white star in d) are microaneurysms. The blood-retinal barrier breakdown manifests as neovascular lesions, which fluoresce brightly and appear blurred as the dye leaks from the vascular lumina (black arrow in b).

Diabetic retinopathy (DR) is one of the most common complications of diabetes mellitus. The surge in diabetes across the globe has led to DR becoming one of the main causes of blindness. Early clinical manifestations of DR include apoptosis of retinal pericytes and increase in permeability of ocular blood vessels. As a consequence, the protective blood-retinal barrier is broken down, which further results in microaneurysm, hard exudates, retinal edema, and minor bleeding. As the disease progresses, the retinal capillary structure becomes so perturbed that all normally perfusable areas are eventually lost (Figure 1). Collectively, these initial clinical changes are diagnosed as nonproliferative (NP) DR, and the disease is considered to have advanced to a proliferative stage (PDR) once revascularization of the affected region has commenced. At this point, successive clinical observations include retinal neovascular proliferation, vitreous hemorrhage, retinal detachment, and loss of vision. Ultimately, the patient succumbs to blindness. Interestingly, retinal edema progression to involve the macula, the central area of the retina, is considered the principal underlying cause of impaired vision.

Many large-population, multicenter clinical studies have been performed to identify the most significant risk factors of DR onset and progression. It appears that the features of diabetes duration, severity of hyperglycaemic episodes, and elevated blood pressure are directly related to DR [1–4]. Many DR pathoincidence theories have been proposed on the basis of these findings, but none have yet to result in an effective “gold-standard” prophylactic or therapeutic strategy. At present, the preferred clinical treatment process is careful maintenance of blood glucose levels and of blood pressure. Events of neovascularization and capillary nonperfusion are commonly treated by partial retinal photocoagulation and pan-retinal photocoagulation, and macular edema is treated by laser and vitrectomy. At the latest stages of DR, retinal reattachment surgery is available.

Despite a vast amount of investigative effort, the pathoincidence of DR is not completely understood. Research findings have implicated roles for the polyol pathway (aldose reductase-mediated conversion of glucose to sorbitol) [5, 6], protein kinase C (a known mediator of glucose transport) [7, 8], advanced glycation end products (AGEs, forming from accumulated glucose), and oxidative stress (a by-product of glucose metabolism) [9, 10]. Moreover, specific immune/inflammatory factors and angioincidence factors [11, 12] have been implicated in the incidence and development of DR [13–18].

The incidence of DR has also gained the interest of clinical geneticists interested in determining whether heredity may significantly contribute to DR risk. Indeed, many DR-susceptibility genes have been identified by their efforts [19–21]. Therefore, DR is believed to have a genetic component, and further research into this mechanism will advance our overall understanding of DR pathogenesis and help to identify targets for potential genome-based therapy.

## 2. ER Stress and DR

**Figure 2:**
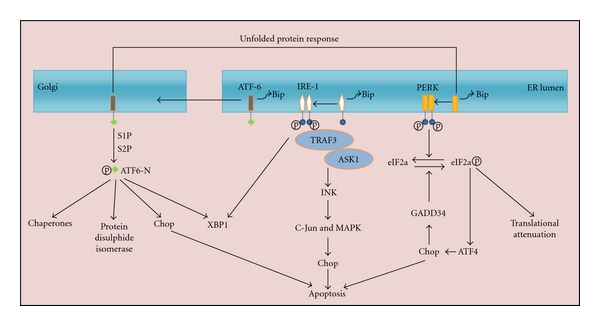
In response to ER stress, Bip separates from the three transmembrane mediators, causing the unfolded protein response to be activated. Unbound PERK then phosphorylates eIF2*α*, leading both to inhibition of new protein translation and to induction of the ATF4 transcription factor. ATF4, in turn, activates CHOP gene expression, which then promotes apoptosis and induces the growth arrest and DNA damage-inducible gene 34 (GADD34). The GADD34 phosphatase dephosphorylates eIF2*α*, thereby completing a negative feedback loop. Meanwhile, the unbound IRE1 initiates splicing of the XBP-1 mRNA. Recruitment of the TNF receptor-associated factor3 (TRAF3) and apoptosis signal-regulating kinase 1 (ASK1) to IRE1 leads to activation of c-Jun amino-terminal kinase (JNK), which in turn activates c-Jun and mitogen-activated protein kinase (MAPK) and ultimately promotes CHOP activity. Unbound ATF6 is cleaved within the Golgi apparatus by the site-1 protease (S1P) and the site-2 protease (S2P) to produce an active transcription factor fragment known as ATF6-N, which in turn activates XBP1 and CHOP; in addition, transcription of ER chaperones and protein disulphide isomerase is increased.

Recently, studies into the underlying molecular mechanisms of DR have suggested that endoplasmic reticulum (ER) stress may play important roles in triggering and maintaining the disease state. The ER organelle mediates processing of newly translated proteins, from synthesis and modification to transport. ER stress is the process of the ER adjusting its function, accelerating or decelerating internal machinery to effectively meet the precise needs of the cell under dynamic conditions. For example, the cellular unfolded protein response (UPR) acts to reduce overall protein synthesis speed, which in turn decreases protein components entering into the ER. Subsequently, expression of the ER molecular chaperones is upregulated, and the protein folding function is accelerated in an attempt for the cell to recover homeostasis. In the event that the UPR becomes too robust or prolonged [22–25], three ER stress factors are induced to facilitate quelling of the process. The pancreatic kinase- (PKR-) like ER kinase (PERK), activating transcription factor 6 (ATF6) and inositol requiring enzyme 1 (IRE1) act by binding to the key ER protein immunoglobulin heavy chain protein/glucose-regulated protein, which has a molecular weight of 78 kDa (Bip/glucose-regulated protein 78 (GRP78)) [26]. However, Bip will dissociate from PERK, ATF6 and IRE1 under conditions of accumulated unfolded or misfolded proteins inside the ER. After such dislocation, the released PERK, ATF6, and IRE1 become activated, and relevant signal transmission is initiated (Figure 2).

A variety of other factors (over 200 known to date) participate in the process of ER stress. These factors have been divided into 11 subclasses, according to their functions: unfolded protein binding, ER protein folding quality control, regulation of cholesterol metabolism, regulation of translation, endoplasmic reticulum-associated degradation (ERAD), ubiquitination, transcription factors, protein folding, protein disulfide isomerization, heat shock proteins (HSPs), and apoptosis [27].

In 2004, Roybal et al. found that activating transcription factor 4 (ATF4), which has been identified as an important factor of ER stress, is capable of directly increasing expression of the vascular endothelial growth factor (VEGF) gene [28]. Since VEGF itself plays an important role in DR, it is possible that ER stress acts through this factor to influence the pathogenesis of DR. In fact, Ikesugi et al. demonstrated that glucose deprivation conditions induced ER stress in retinal pericytes [29], supporting the notion that ER stress participates in incidence and development of DR. Oshitari et al. also found that ER stress was involved in ocular vascular abnormalities in human DR patients [30, 31]. Further experimental investigation in animal models of diabetes and oxygen-induced retinopathy (OIR), carried out by Zhang et al., demonstrated that ER stress was activated in the affected retinas and indicated that ER Stress is a potential mediator of retinal inflammation in DR [32]. The authors also demonstrated that ER stress preconditioning could protect against retinal endothelial inflammation through activation of X-box-binding protein (XBP)1-mediated UPR and inhibition of NF-*κ*B activation [33].

ER stress is well known to elicit induction effects on inflammatory factors, and inflammation is believed to play a critical role in DR; thus, many researchers are currently exploring the effects of ER stress on DR via the actions of inflammatory factors [34]. Our laboratory also focuses on this mechanism of DR.

## 3. Identifying DR-Susceptibility and DR-Protective Genes

The bulk of research on DR hereditary factors performed to date has focused on patients with preexisting illness (i.e., diabetes), complicating the discovery of true DR-susceptibility genes. Yet, it has been clearly observed that the occurrence and severity of DR in diabetic patients varies among individuals. Some patients develop DR relatively soon after their diabetes diagnosis, while others do not develop DR for decades. In the same sense, some cases of DR are mild, while others are severe. Neither the aggressiveness nor severity of DR have been related to control of blood sugar levels or extent of loss of control, further indicating an underlying role for genetic susceptibility. Therefore, it appears that some diabetic patients have an inherent resistance to developing the DR complication or are better equipped to limit its pathogenesis.

It has been estimated that up to 20% of patients who have suffered from diabetes for 20 years remain free of the DR complication [35]. Since there are no medicines available which can effectively control DR and mitigate its progression, it can be concluded that expression of a particular gene or set of genes in these DR-resistant patients can help to protect against diabetes/glucose-related injury to retinal vessels. Even if these genes do not protect patients from DR over their entire lifetime, they may substantially delay onset or lessen severity of DR. Therefore, identification of these genes and gaining a detailed understanding of their expression patterns will likely lead to development of new therapeutic targets for genetic-based DR treatment.

On the basis upon previously published study designs for detecting genes associated with a disease state [36, 37], we chose a cohort of Type 2 diabetes patients (*n* = 59) with long-standing diagnosis (20 years or more). These patients were divided into two groups: not complicated by DR (normal) and complicated by DR. Gene expression analysis was performed by microarray (GeneChip human genome U133 plus 2.0; Affymetrix, Santa Clara, Calif, USA), and statistically significant differences in expression profiles were determined by comparative analysis among the two groups. Careful analysis of the enrolled patients' demographics led to exclusion of 22 patients from the normal group and 37 patients from PDR group. From the remaining patients, 20 were selected from each group, and venous blood samples were obtained for total RNA extraction. The RNAs of six patients from each group were preferentially selected to conduct gene chip detection. We found that diabetic patients without DR complication presented with 173 overexpressed genes (*P* < 0.05), compared to the PDR group [38]. These differential expression results were confirmed by quantitative PCR.

Thereafter, the functions of these 173 genes were analyzed, and it was found that 46 were related to protein degradation and structure modification. In addition, several factors known to be involved in the ER stress process were also found. Thus, we concluded that these genes that were suppressed in PDR patients may represent genes that provide a protective effect against DR.

## 4. The Role of P58^IPK^ in DR

One of the differentially expressed genes in our study of non-DR diabetic patients versus PRD-afflicted diabetic patients that piqued our interest was P58^IPK^. This gene encodes a 58 kDa inhibitor of the interferon-induced double-stranded RNA-activated protein kinase (also known as DNAJC3) and is a member of the Hsp40 family. First characterized for its activities as an inhibitor of the key translation-mediator eukaryotic initiation factor 2*α* (eIF2*α*) [39], P58^IPK^ has since been determined to play an essential role in preventing ER stress [40, 41]. The mechanism by which P58^IPK^ affects ER stress was determined to involve inhibition of PERK activation [42, 43], suggesting that P58^IPK^ acts as a key mediator of cotranslocational ER protein degradation; moreover, this process is likely to contribute to ER homeostasis in stressed cells. Mutant mouse strains with P58^IPK^ gene deletion displayed glucosuria, hyperglycemia, and hypoinsulinemia [44], suggesting that P58^IPK^ plays important roles in maintaining normal glucose levels. P58^IPK^ must enter the endoplasmic reticulum through a translocon in order to perform its protein synthesis and folding functions. Interestingly, P58^IPK^ can also prevent a polypeptide chain from entering the ER through the translocon, thereby reducing protein loading of the ER and protecting cells from the stress state [45, 46]. When polypeptide chains are excluded from the ER, they are subject to degradation by the ubiquitin system. Accordingly, it has been reported that ER stress can be experimentally induced in rat pancreatic *β* cells by eliminating P58^IPK^ as a result, the islet cells of these rats experience significant apoptosis and develop diabetes [47].

We were the first to investigate the effect of P58^IPK^ on DR by performing extracorporeal experiments. Human retinal capillary endothelial cells (HRCECs) were cultured *in vitro* and transfected with a P58^IPK^ overexpressing vector or P58^IPK^ RNA interference (RNAi) to suppress expression. As expected, P58^IPK^ expression was significantly increased in cells transfected with adeno-associated virus vector- (rAAV2-)  P58^IPK^ (0.63 ± 0.02), as compared to those transfected with pGIPZ-P58^IPK^ RNAi (0.23 ± 0.01). P58^IPK^ expression was not different between the control transfected cells (rAAV2-GFP and pGIPZ-GFP). ER stress was induced in the transfected cells by treating with tunicamycin and changes in the expression of P58^IPK^ were determined, along with that of VEGF, core/emopamil-binding protein (C/EBP) homologous protein (CHOP), ATF4, and GRP78. Apoptosis levels were also determined for the P58^IPK^ overexpressing cells and suppressed cells. ER stress had no effect on gene expression in cells overexpressing P58^IPK^, as evidenced by no difference in expression levels of ATF-4, GRP78, CHOP, and VEGF as compared to those in unstressed control cells. However, the inhibitory effect of P58^IPK^ on the expression of ER stress-related factors was suppressed in cells transfected with pGIPZ-  P58^IPK^ RNAi. Apoptosis was also found to be significantly increased in cells transfected with pGIPZ-P58^IPK^ RNAi and not in those transfected with rAAV2-P58^IPK^ [48].

We also investigated the effects of P58^IPK^ overexpression on the retinas of rats with sustained high glucose. A rat diabetic model was established by intraperitoneal injection of streptozotocin. Overexpression of P58^IPK^ was achieved by intravitreal injection of purified recombinant rAAV2-P58^IPK^ or transfection into cultured rat retinal capillary endothelial cells. Retinal vascular permeability was determined by assessing the Evans Blue retinal leakage. To downregulate the P58^IPK^ level in cultured rat retinal capillary endothelial cells, pGIPZP58^IPK^ RNAi was introduced in these cells. Real-time reverse transcription- (RT-) PCR and Western blot analyses were performed to evaluate the mRNA and protein levels, respectively, of CHOP, VEGF, and tumor necrosis factor-*α* (TNF-*α*). Results showed that retinal blood vessel leakage was significantly decreased in diabetic rats overexpressing P58^IPK^, as compared with the control diabetic rats. Both mRNA and protein levels of CHOP, TNF-*α*, and VEGF were remarkably reduced in the retinas of diabetic rats overexpressing P58^IPK^. *In vitro* study further demonstrated that overexpression of P58^IPK^ led to the downregulation of CHOP, TNF-*α*, and VEGF gene expression under high glucose conditions, whereas RNAi suppression of P58^IPK^ enhanced the expression of CHOP, TNF-*α*, and VEGF [49].

Collectively, these studies indicated that P58^IPK^ functions include protecting the integrity of retinal vessels and resisting development and progression of DR. Its key role as a stabilizing endoplasmic reticulum factor led us to presume that P58^IPK^ contributes to DR by reducing incidence of ER stress through maintaining stability of the ER; these studies are underway. Nonetheless, P58^IPK^ appears to be a particularly promising genetic target to develop therapy to protect against DR. 

## 5. Which ER Stress-Related Factors Protect against DR?

Research by our group and others are continuing to investigate the contribution of the full panel of ER stress-related factors to DR. We established a high glucose rat model in order to observe the expression changes of these ER stress-related factors. At the same time, Ikesugi and colleagues and Roybal and colleagues [28, 29] reported that in the early stages of high glucose in rats the expressions of VEGF and CHOP were upregulated, but the expressions of GRP78 and ATF4 remained stable. Again, the animal experiment results were consistent with those from the extracorporeal system [50]. Therefore, the studies to determine exactly which ER stress factors play critical roles in resisting DR and the mechanisms by which they act are of clinical interest.

**Figure 3:**
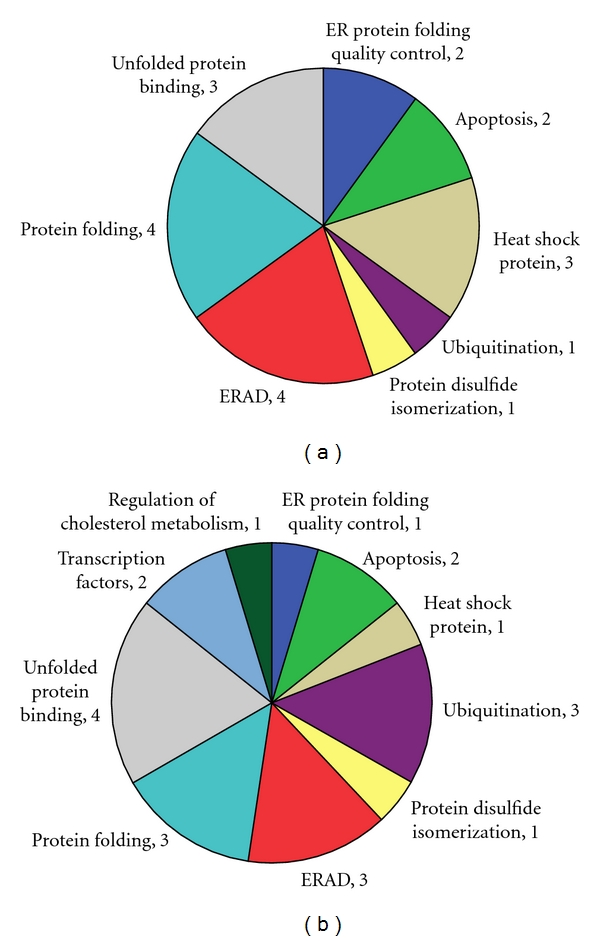
Associated functions of ER stress-related factors differentially expressed in diabetic retina in the first (a) and third (b) month after development of diabetes (numbers of proteins identified for each function are indicated). Expression levels were determined by quantitative real-time RNA polymerase chain reaction microarrays and compared to those of normal nondiabetic rats; differential expression was designated if a gene was detected at 2-fold lower levels.

It is ultimately necessary to gain a comprehensive understanding of the effects of ER stress-related factors on DR. To this end, we selected 89 factors from the entire panel of known ER stress-related factors representing each of the 11 subclasses of function in ER stress, [27] and on the basis of relevant studies of ER stress from the literature [28, 32, 51–69]. Custom-made real-time PCR chips based on the rat sequences for these 89 genes were designed (SABiosciences, Gaithersburg, Md, USA) and employed for accurate detection of temporal expression changes of these factors in the retina of high glucose rats. The results indicated that 13 genes, including the P58^IPK^ gene, were significantly down-regulated in the high glucose rat model at 1 month old. In three-month-old high glucose rats, 12 genes were downregulated (Figure 3). In addition, we found that three key signaling pathways of ER stress (PERK, IRE1 and ATF6) were not activated in the early stage of high glucose in these rats. This phenomenon is consistent with our earlier research findings [50]. According to the results of our gene chip studies, the ERAD pathway-related factors were of particular interest. The effects of P58^IPK^ and ERAD in ER stress were discussed in this paper. Firstly, both have effects on maintaining functional balance of the endoplasmic reticulum and in preventing ER stress. P58^IPK^ mediates the transport of new unfolded proteins entering into the endoplasmic reticulum [45], while ERAD degrades the unfolded proteins that have accumulated in the endoplasmic reticulum. Removal (processing) of unfolded proteins from the endoplasmic reticulum is crucial to prevent ER stress, and is a more direct control mechanism than the transport modulation by P58^IPK^. Therefore, we have theorized that overexpression of ERAD-related factors in the retina might be able to boost the ERAD signaling pathways to a more robust level and prevent ER stress under high glucose conditions and halt or slow down the progress of DR.

## 6. Perspectives for Future Studies

Much like the information gleaned from studies to identify disease-susceptibility genes, data on disease-protective genes provide the foundational knowledge by which our understanding of human health is advanced and effective healthcare strategies are developed. If the pathological mechanism of DR is the proverbial “black box”, then defining the gene expression profile of DR will represent a window through which we may observe and assess the situations inside of it. Using this idea as a guide to our own studies, we have gained significant insights into which ER stress-related factors participate in the onset and pathogenic process of DR and identified a promising target for DR treatment.

However, like most disease processes, DR involves many signaling pathways and physiological factors. Cross-talk and functional interactions among these factors certainly form a complex and dynamic network structure. Each individual gene that is characterized as having a protective role against DR effectively represents a single node in that entire network. Therefore, the collective analysis of the DR gene expression profile must be continued and augmented with studies to understand the influence of clinical (comorbidities) and nonphysiologic aspects (environmental factors).
